# Phosphatidylinositol 4-Kinase IIIβ: A Therapeutic Target for Contractile Dysfunction in Hypertrophic Cardiomyocytes

**DOI:** 10.3390/ijms27020595

**Published:** 2026-01-07

**Authors:** Myrthe M. A. Willemars, Aomin Sun, Shujin Wang, Ozlenen Simsek Papur, Agnieszka Brouns-Strzelecka, Rick van Leeuwen, Sabina J. V. Vanherle, Dimitrios Kapsokalyvas, Jan F. C. Glatz, Dietbert Neumann, Miranda Nabben, Joost J. F. P. Luiken

**Affiliations:** 1 Department of Genetics & Cell Biology, Faculty of Health, Medicine and Life Sciences, Maastricht University, 6229 ER Maastricht, The Netherlands; 2 Cardiovascular Research Institute Maastricht (CARIM), 6229 ER Maastricht, The Netherlands; 3 Department of Physiology, Faculty of Health, Medicine and Life Sciences, Maastricht University, 6229 ER Maastricht, The Netherlands; 4 Beijing Key Laboratory of Gene Resource and Molecular Development, College of Life Sciences, Beijing Normal University, Beijing 100875, China; 5 Key Laboratory of Cell Proliferation and Regulation Biology, Ministry of Education, College of Life Sciences, Beijing Normal University, Beijing 100875, China; 6 Institute of Life Sciences, School of Basic Medicine, Chongqing Medical University, Chongqing 400016, China; 7 Department of Molecular Medicine, Institute of Health Science, Dokuz Eylul University, 35210 Izmir, Turkey; 8 Department of Cardiology, Faculty of Health, Medicine and Life Sciences, Maastricht University, 6229 ER Maastricht, The Netherlands; 9 Department of Clinical Genetics, Maastricht University Medical Center+, 6229 HX Maastricht, The Netherlands; 10 Interdisciplinary Center for Clinical Research (IZKF), University Hospital RWTH Aachen, 52074 Aachen, Germany; 11 Department of Pathology, Maastricht University Medical Center+, 6229 HX Maastricht, The Netherlands

**Keywords:** cardiac metabolism, phosphatidylinositol 4-kinase IIIβ, cardiac hypertrophy, contractile dysfunction, heart failure

## Abstract

Cardiac hypertrophy is an important risk factor for heart failure and is often accompanied by contractile dysfunction. While hypertrophic growth contributes to disease progression, the underlying molecular mechanisms remain incompletely understood. A proposed contributor is a metabolic shift toward glucose uptake, suggesting that kinases regulating this process, such as protein kinase D1 (PKD1) and downstream target phosphatidylinositol 4-kinase IIIβ (PI4KIIIβ), might be effective targets to mitigate cardiac hypertrophy-induced contractile dysfunction. We investigated whether PI4KIIIβ inhibition downregulates enhanced glucose uptake in hypertrophic cardiomyocytes and thereby treats cardiac hypertrophy-induced contractile dysfunction. Hypertrophy was induced in cultured adult rat cardiomyocytes and human stem cell-derived cardiomyocytes using either phenylephrine (PE) or adenoviral PKD1 overexpression. PE-induced hypertrophy was associated with increased mRNA expression of BNP, activation of hypertrophic signaling, morphological alterations, enhanced protein synthesis and glucose uptake, and impaired contractile function. Treatment with the PI4KIIIβ inhibitor MI14 prevented and reversed PE-stimulated glucose uptake and contractile dysfunction, while hypertrophic signaling, cell size, and protein synthesis remained unaffected. Similar effects on glucose uptake were observed in the PKD1 overexpression model. These findings suggest that targeting myocardial substrate metabolism via the PI4KIIIβ pathway, rather than hypertrophic growth itself, could be a promising strategy to treat hypertrophy-induced contractile dysfunction.

## 1. Introduction

Cardiovascular disease (CVD) is the number one cause of death globally, with cardiac hypertrophy as the main reason of mortality among patients who have CVD [1,2]. Cardiac hypertrophy provides short-term benefits, such as reducing wall stress and sustaining cardiac function, but in the long-term hypertrophy is often followed by contractile dysfunction and eventually heart failure [2,3]. Therefore, it is urgent to develop prevention strategies and therapies to treat cardiac hypertrophy. At the cellular level, cardiac hypertrophy is characterized by an increase in cardiomyocyte size, enhanced protein synthesis, metabolic changes and re-induction of the fetal gene program [4,5]. The metabolic shift to an increase in glucose uptake has been considered an important contributor in the development of cardiac hypertrophy and concomitant heart failure [6,7,8,9]. Therefore, strategies to downregulate glucose uptake could be an attractive way to prevent and/or regress cardiac hypertrophy-induced contractile dysfunction.

Protein kinase D1 (PKD1) is a key player in the development of cardiac hypertrophy and has been studied as a potential target to restore cardiac function in the hypertrophied heart [10,11]. Sustained mechanical stress such as pressure overload results in PKD1 activation, and subsequent phosphorylation of its direct target histone deacetylase 5 (HDAC5). In the non-phosphorylated state, HDAC5 interacts with the transcription factor myocyte enhancer factor-2 (MEF2). Upon HDAC5 phosphorylation, MEF2 is released and drives hypertrophic programming [12]. Knockdown and inhibition of PKD1 suppress hypertrophy of the heart and of cardiomyocytes, respectively [13,14]. In addition to stimulation of hypertrophic programming, PKD1 plays a key stimulatory role in cardiac glucose uptake, as established in vitro [15] and in vivo [16], as it affects GLUT4 translocation from intracellular stores to the cell surface.

Given that PKD1 is positioned at the crossroad between stimulation of cardiac glucose uptake and hypertrophic signaling and that hypertrophy can be beneficial short-term, we sought to look downstream of PKD1 and to directly target cardiac metabolism rather than cardiac hypertrophy. Our previous studies have yielded the identification of phosphatidylinositol 4-kinase IIIβ (PI4KIIIβ), a direct downstream target of PKD1 [17]. This lipid kinase proved to be a central positive player in stimulation of glucose uptake in cardiomyocytes by mediating the assembly of the GLUT4 translocation machinery. Importantly, this kinase appeared not to be involved in hypertrophic signaling [17], making it a more specific target to cure the hypertrophic heart via downregulation of glucose uptake.

In this study, we aimed to investigate whether selective downregulation of enhanced glucose uptake in hypertrophic cardiomyocytes via inhibition of PI4KIIIβ can prevent and treat hypertrophy and hypertrophy-induced contractile dysfunction. We applied an in vitro model of hypertrophy via α-adrenergic stimulation with phenylephrine (PE) in adult rat cardiomyocytes (as previously established [18,19]) and in human stem cell-derived cardiomyocytes. This in vitro PE model showed the well-characterized major abnormalities of the hypertrophic heart: increased mRNA expression of the hypertrophic marker brain natriuretic peptide (BNP) [20,21,22], enhanced phosphorylation of hypertrophy-related signaling kinases including mammalian target of rapamycin complex 1 (mTORC1), extracellular signal-regulated kinase 1/2 (ERK1/2), cardiac contractile protein troponin I (TnI) and PKD1/HDAC5 [23], increased protein synthesis [4,24,25], enhanced glucose uptake [26,27,28,29,30], and impaired contractile function [31]. To investigate whether inhibition of PI4KIIIβ would downregulate glucose uptake in hypertrophic cardiomyocytes and hypertrophy-induced contractile dysfunction, we co-treated adult rat cardiomyocytes with a specific PI4KIIIβ inhibitor: MI14. This compound was selected from a series of compounds that have little affinity towards the closely related family member PI4KIIIα, or to the more distant PI4KIIα [32]. The specific PI4KIIIβ inhibitor MI14 showed to prevent and treat PE-stimulated glucose uptake and PE-induced contractile dysfunction in adult rat cardiomyocytes, while fatty acid uptake, hypertrophic signaling, gene expression and protein synthesis remained unchanged. MI14’s ability to prevent increased glucose uptake was also confirmed in another aRCM hypertrophy model (adenoviral PKD1 overexpression) and in PE-stimulated human stem cell-derived cardiomyocytes.

## 2. Results

### 2.1. MI14 Prevents Phenylephrine-Induced Glucose Uptake in Adult Rat Cardiomyocytes

**Figure 1:**
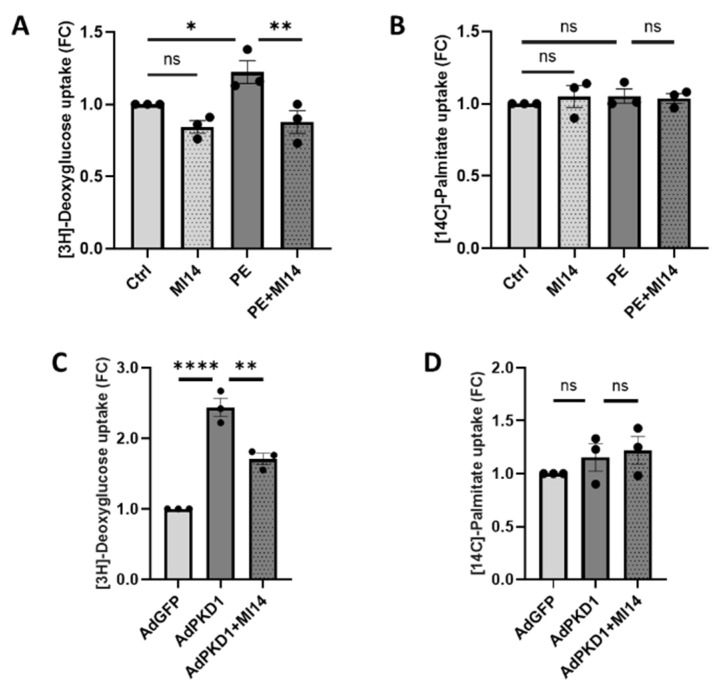
MI14 blocks phenylephrine (PE) and adenoviral PKD1 (AdPKD1)-overexpression stimulated glucose uptake in isolated adult rat cardiomyocytes (aRCM). (**A**,**B**) Isolated aRCM was incubated with either no stimulation, MI14, PE or PE together with MI14 for 24 h. (**A**) Uptake of [^3^H]-deoxyglucose by aRCM (*n* = 3). (**B**) Uptake of [^14^C]-palmitate by aRCM (*n* = 3). (**C**,**D**) Isolated aRCM was transduced with AdGFP or AdPKD1, followed by 24 h incubation without treatment or with MI14. (**C**) Uptake of [^3^H]-deoxyglucose by aRCM (*n* = 3). (**D**) Uptake of [^14^C]-palmitate by aRCM (*n* = 3). Data is reported as fold change of control ± SEM. * *p* < 0.05, ** *p* < 0.01, **** *p* < 0.0001. FC, fold change; PE, phenylephrine.

We previously established an in vitro cardiac hypertrophy model, using isolated adult rat cardiomyocytes treated with 50 µM PE for 24 h [18]. Here, we co-incubated cardiomyocytes during PE stimulation with 2.5 µM of the PI4KIIIβ inhibitor MI14 for 24 h. This did not affect cell viability (Figure S2). We then examined the effect of MI14 on PE-induced glucose and fatty acid uptake. PE stimulation of cardiomyocytes increased glucose uptake (Figure 1A), in line with our previous findings [18]. This increase in glucose uptake was prevented when cells were co-treated with MI14 (Figure 1A). Meanwhile, fatty acid uptake remained unchanged (Figure 1B). The same inhibitory action on glucose uptake by MI14 was found in an adenoviral PKD1 (AdPKD1) overexpression model that is known to increase GLUT4 translocation and glucose uptake, as well as induce hypertrophy (Figure 1C,D) [33]. In conclusion, MI14 prevents increased glucose uptake in hypertrophic cardiomyocytes.

### 2.2. MI14 Does Not Prevent Phenylephrine-Induced Hypertrophic Signaling, Protein Synthesis and BNP Expression in Adult Rat Cardiomyocytes

**Figure 2:**
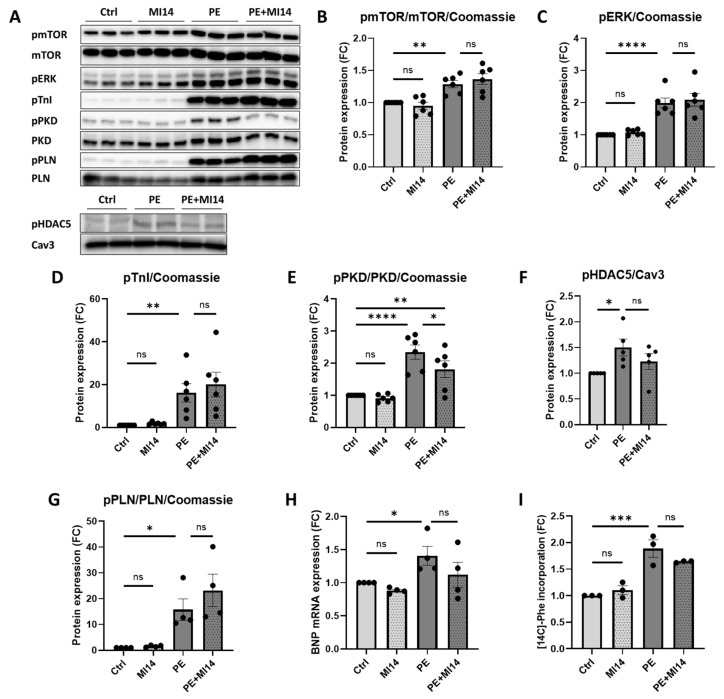
MI14 does not affect phenylephrine (PE)-stimulated hypertrophic signaling, gene expression and protein synthesis in adult rat cardiomyocytes (aRCM). (**A**) Representative Western blots of phosphorylated and total mTOR, ERK, TnI, PKD, HDAC5 and PLN. (**B**–**G**) Quantification of target proteins normalized to Coomassie (see Figure S3) or caveolin 3 (*n* = 3–6). (**H**) mRNA expression of BNP in aRCM (*n* = 4). (**I**) Protein synthesis in aRCM expressed as [^14^C]-phenylalanine incorporation (*n* = 3). Data is reported as fold change of control ± SEM. * *p* < 0.05, ** *p* < 0.01, *** *p* < 0.001, **** *p* < 0.0001. BNP, brain natriuretic peptide; Cav3, caveolin 3; ERK, extracellular signal-regulated kinase; FC, fold change; HDAC5, histone deacetylase 5; mTOR, mammalian target of rapamycin; PE, phenylephrine; Phe, phenylalanine; PKD, protein kinase D1; PLN, phospholamban; TnI, troponin I.

To unravel the effect of MI14 on the signaling pathways involved in the development of cardiac hypertrophy, we examined phosphorylation levels of the hypertrophy-associated kinases mTOR, ERK, PKD, the MEF2 adaptor HDAC5 and the Ca^2+^ dynamics regulator phospholamban (PLN) [34]. We also measured phosphorylation of cardiac contractile protein TnI, which is considered a biomarker for chronic heart failure, including hypertrophy-induced cardiomyocyte dysfunction [35]. As expected, each of the phosphorylation levels of mTOR, ERK, TnI, PKD, HDAC5 and PLN was increased in PE-stimulated cardiomyocytes, supporting the use of PE stimulation as a suitable in vitro model of cardiac hypertrophy. Yet, none of these increased phosphorylation events were prevented by co-incubation of cells with MI14 (Figure 2A–G). Notably, co-incubation with MI14 did decrease but not prevent PKD phosphorylation (Figure 2E). However, 15 min MI14 co-incubation after 24 h PE stimulation did not affect PKD phosphorylation (Figure S4). This suggests a potential positive feedback loop where PI4KIIIβ activation further enhances PKD activity, which may be suppressed by MI14.

To further confirm the lack of an effect of MI14 on the development of hypertrophy in cardiomyocytes, we assessed mRNA levels of the hypertrophy marker BNP and protein synthesis in PE-treated cardiomyocytes. As expected, PE stimulation increased mRNA expression of BNP and protein synthesis, but MI14 showed no influence on these parameters (Figure 2H,I). Taken together, these results indicate that PI4KIIIβ inhibition by MI14 does not affect PE-induced hypertrophy.

### 2.3. MI14 Prevents Phenylephrine-Induced Contractile Dysfunction in Adult Rat Cardiomyocytes

**Figure 3:**
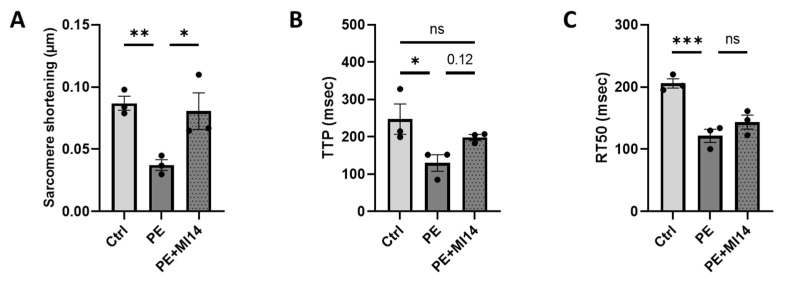
MI14 prevents phenylephrine (PE)-induced contractile dysfunction in adult rat cardiomyocytes (aRCM). Contractile properties were assessed under 30 V 1 Hz electric-field stimulation. (**A**) Sarcomere shortening, (**B**) time to peak (TTP) and (**C**) decay time (RT50) of aRCM (*n* = 3). Data is reported as mean ± SEM. * *p* < 0.05, ** *p* < 0.01, *** *p* < 0.001. PE, phenylephrine.

To elucidate whether MI14 could protect adult rat cardiomyocytes from PE-induced contractile dysfunction, we measured contractile properties during 1 Hz electric field stimulation. PE stimulation decreased sarcomere shortening, the time to reach maximal contraction (TTP) and the decay time to 50% relaxation (RT50) (Figure 3). This decrease in sarcomere shortening demonstrates that PE stimulation resulted in cardiomyocyte contractile dysfunction. Co-incubation with MI14 restored the PE-induced decrease in sarcomere shortening and improved contraction acceleration time, without a change in relaxation time. Thus, co-incubation of MI14 prevents PE-induced contractile dysfunction. Additionally, MI14 did not affect basal contractile function (Figure S5).

### 2.4. MI14 Not Only Prevents, but Also Restores Glucose Uptake and Contractile Function in Adult Rat Cardiomyocytes

**Figure 4:**
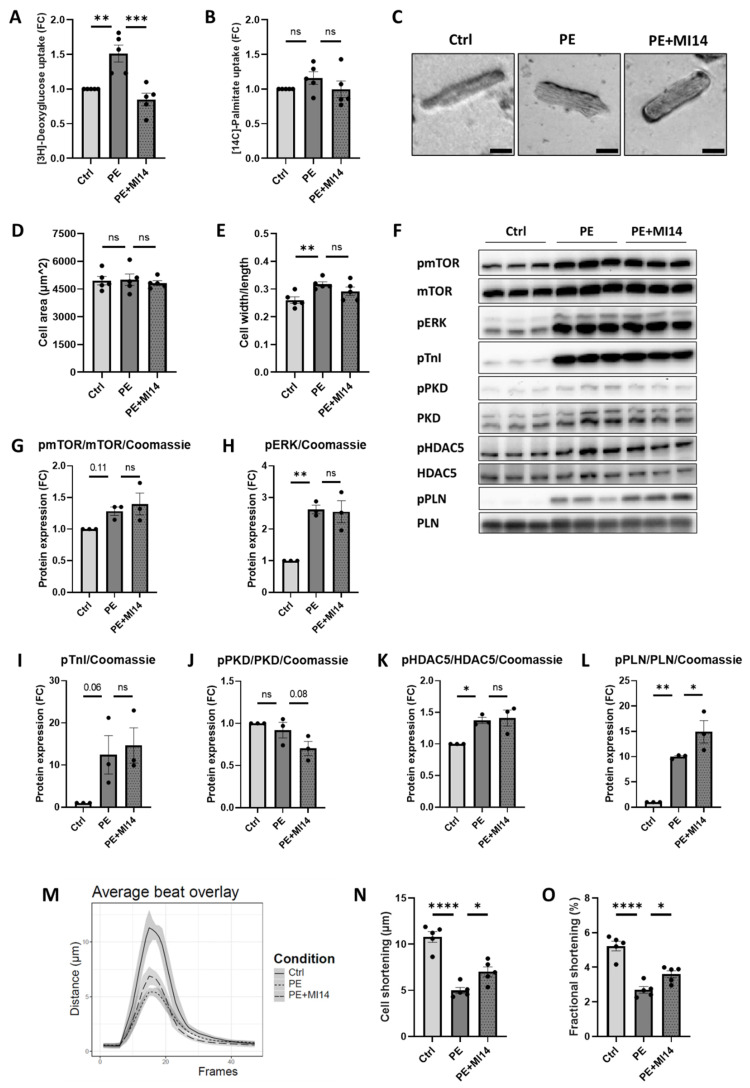
MI14 treats increased glucose uptake and contractile dysfunction without affecting hypertrophy in adult rat cardiomyocytes. Isolated aRCM were incubated with or without PE for the first 24 h to induce hypertrophy, followed by 24 h of either no stimulation, PE or PE together with MI14. (**A**) Uptake of [^3^H]-deoxyglucose by aRCM (*n* = 5). (**B**) Uptake of [^14^C]-palmitate by aRCM (*n* = 5). (**C**) Representative images of cell dimensions. Scale bar is 50 µm. (**D**,**E**) Cardiomyocyte size expressed in cell area (7–10 cells/condition/*n* with *n* = 5) and cell width/length (see Figure S6) (9–11 cells/condition/*n* with *n* = 5). (**F**) Representative Western blots of phosphorylated and total mTOR, ERK, TnI, PKD, HDAC5 and PLN. (**G**–**L**) Quantification of target proteins normalized to Coomassie (see Figure S3) (*n* = 3). (**M**) Cardiomyocyte contraction traces. (**N**,**O**) Contractile function of aRCM expressed as cell shortening or fractional shortening (*n* = 5). Data is reported as mean or fold change of control ± SEM. * *p* < 0.05, ** *p* < 0.01, *** *p* < 0.001, **** *p* < 0.0001. ERK, extracellular signal-regulated kinase; FC, fold change; HDAC5, histone deacetylase 5; mTOR, mammalian target of rapamycin; PE, phenylephrine; PKD, protein kinase D1; PLN, phospholamban; TnI, troponin I.

After demonstrating the capabilities of PI4KIIIβ inhibition to prevent metabolic and associated contractile changes, we determined the ability of MI14 to exert beneficial effects on cardiomyocytes that are already hypertrophic. Therefore, adult rat cardiomyocytes were first made hypertrophic by 24 h PE stimulation, followed by 24 h co-incubation of PE with MI14. As expected, PE stimulation for 48 h significantly increased glucose uptake and decreased absolute/fractional cell shortening, while MI14 co-incubation for the last 24 h restored glucose uptake and improved contractile function (Figure 4A,M–O). On the other hand, fatty acid uptake was not affected by PE stimulation nor by MI14 co-treatment (Figure 4B), in agreement with the prevention experiments (Figure 1). Moreover, the stimulatory effects of PE on hypertrophic signaling (Figure 4F–K) and adaptations in cellular dimensions (Figure 4C–E and Figure S4) were also unchanged by MI14 co-incubation. Of note, and in contrast to the increased PE-induced phosphorylations of mTOR, pERK, pTnI, HDAC5 and PLN, PKD phosphorylation (found to be increased after 16 h and 24 h (Figure 2E) [18]) was not increased anymore after 48 h (Figure 4J). This decrease in PE-induced PKD activation over time has been observed previously [18], which may be due to a feedback loop in hypertrophic signaling. In addition, PLN phosphorylation was further increased after co-incubation with MI14 (Figure 4F,L).

### 2.5. MI14 Lowers Phenylephrine-Induced Glucose Uptake in Human Stem Cell-Derived Cardiomyocytes

**Figure 5:**
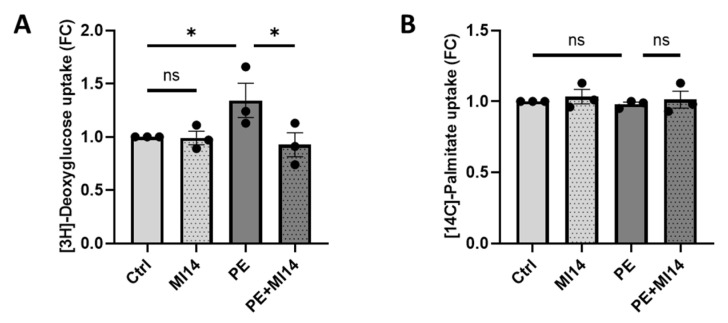
MI14 lowers PE-induced glucose uptake in iPSC-CM, while fatty acid uptake remains unchanged. (**A**) Uptake of [^3^H]-deoxyglucose by iPSC-CM (*n* = 3). (**B**) Uptake of [^14^C]-palmitate by iPSC-CM (*n* = 3). Data is reported as fold change of control ± SEM. * *p* < 0.05. FC, fold change; PE, phenylephrine.

In order to investigate the potential beneficial effects of PI4KIIIβ inhibition in human cardiomyocytes when exposed to hypertrophic stimulation, iPSC-CM was treated with PE and/or MI14 for 24 h after which glucose and fatty acid uptake was measured. As with aRCM, PE incubation resulted in increased glucose uptake, while co-incubation of PE with MI14 prevented the increase in glucose uptake (Figure 5A). Meanwhile, fatty acid uptake remained unchanged (Figure 5B).

## 3. Discussion

In the hypertrophic heart, there is a metabolic change in which the heart increasingly utilizes glucose, making it the main energy source instead of fatty acids [36,37,38,39,40]. This is thought to contribute to the further development of cardiac hypertrophy and subsequently heart failure [6,7,8,9]. Therefore, downregulation of glucose uptake could be an interesting strategy to prevent and/or treat cardiac hypertrophy and cardiac hypertrophy-induced contractile dysfunction. In this study, we used PE-stimulated cardiomyocytes (from both rat and human origin) as an in vitro cardiac hypertrophy model and investigated whether inhibition of PI4KIIIβ by MI14 is able to prevent and/or reverse the development of cardiac hypertrophy-associated contractile dysfunction. We found that PI4KIIIβ inhibition via MI14 is not only able to prevent but also to reverse a metabolic shift towards increased glucose uptake and associated contractile dysfunction, while hypertrophic signaling, gene expression and protein synthesis remained unchanged.

The ability of MI14 to inhibit PE-stimulated glucose uptake indicates the involvement of PI4KIIIβ. This is a novel observation, given that to the best of our knowledge PI4KIIIβ has not been considered in the signaling effects of PE so far. Nevertheless, the involvement of PI4KIIIβ in PE-stimulated glucose uptake could be expected, because this is in line with our previous findings that PI4KIIIβ is downstream of PKD1 in cardiomyocytes upon activation of contraction signaling and contraction-activated glucose uptake [17]. PKD1 is not only a contraction-activated kinase [15], but also for a longer time known to be involved in cardiac hypertrophy [41]. Hence, the PKD1–PI4KIIIβ axis is involved in both contraction-stimulated glucose uptake and hypertrophy-associated glucose uptake. The phosphorylation of PI4KIIIβ by PKD1, as reported at Ser294, will lead to binding of the adaptor protein 14-3-3 [17,42]. Beyond recruitment of 14-3-3, the sequence of events towards GLUT4 translocation is not completely elucidated, but there is evidence that 14-3-3 brings PI4KIIIβ to a cytoplasmic pool of phosphatidylinositol (PI) close to the endosomes and bound to PI transfer proteins (PITP). This event allows PI4KIIIβ to gain access to its substrate, and upon completion of the catalytic conversion of PI into PI4P, PI4P will be expelled from the PITPs to form specific membrane domains within the endosomes. These PI4P-enriched domains will then serve as platforms for the recruitment of proteins (perhaps including clathrins) making up the GLUT4 translocation machinery [11].

Besides prevention/reversal of maladaptive stimulation of GLUT4-mediated glucose uptake, MI14 treatment also prevented and reversed contractile dysfunction in the hypertrophic cardiomyocytes. The link between these beneficial MI14 effects on glucose uptake and contractile function corresponds with accumulating evidence that a shift of substrate preference towards glucose in the hypertrophic heart contributes to the progression of contractile dysfunction [43]. Several studies show that strategies to shift the cardiac substrate utilization back to fatty acids by specifically increasing fatty acid utilization or decreasing glucose utilization in the hypertrophic heart can be protective against contractile dysfunction [44]. For example, high-fat diet attenuates contractile dysfunction in rats with cardiac hypertrophy [45]. Reduction in glucose uptake via propranolol preserves cardiac function in mice with pressure overload-induced left ventricular hypertrophy [7]. Hence, our present observations of MI14-mediated inhibition of glucose uptake and preservation of contractile function are in line with these previous studies. Yet, in contrast to these studies in which a high-fat diet and propranolol administration induce many other cellular changes besides reduction in glucose uptake, the present pharmacological approach is much more directed to specific inhibition of glucose uptake.

Surprisingly, the MI14-mediated preservation of contractile function did not depend on preventing PE-stimulated mTOR activation coupled to preserving low rates of protein synthesis. Such similar dissociation between contractile function (which was preserved) and protein synthesis (increased) was also found in our previous studies in PE-stimulated cardiomyocytes in which GLUT4 translocation and glucose uptake were inhibited by dipyridamole [18,46]. Yet, in that previous study, it was more problematic to couple inhibition of GLUT4-mediated glucose uptake to contractile preservation because dipyridamole is also acting as a phosphodiesterase inhibitor and nucleoside transport inhibitor unrelated to glucose uptake [47,48].

The beneficial action of MI14 on contractile function was not only independent of mTOR and protein synthesis, but also appeared unrelated to the HDAC5–MEF2 pathway. Namely, the lack of inhibition of HDAC5 phosphorylation upon MI14 treatment indicates that the hypertrophic transcription factor MEF2 is in its free form to enter the nucleus and activate the hypertrophic genes under its control. Additionally, the lack of inhibition of ERK1/2 phosphorylation suggests that ERK remains in the nucleus in an activated state to phosphorylate and activate several transcription factors other than MEF2, such as CREB and Elk1 [49]. Yet, despite the fact that all these hypertrophic transcription factors are presumably in a maladaptive unimpaired state during MI14 treatment, contractile function is rescued. Taken together, these findings suggest that reduction in glucose uptake is a suitable strategy to preserve contractile function in the hypertrophic heart. This supports the overall concept that cardiac disease in general may be prevented and even treated by metabolic interventions. We have yet to determine the effects of prolonged MI14 treatment, exceeding 24 h. Potentially upon prolonged treatment, the other hypertrophy-induced processes as mentioned above (increased protein synthesis, Ca^2+^ dynamics and hypertrophic gene programming) will be reduced to basal levels, as secondary effects following reduced glucose uptake. Finally, the lack of a direct effect of MI14 on phosphorylation of PKD1/ERK/mTOR/TnI provides firm evidence in favor of the specificity of MI14 towards PI4KIIIβ in a cellular context next to the previously performed cell-free assays in which limited affinity was shown towards other PI4K members (see Section 1).

## 4. Materials and Methods

### 4.1. Animals

Male Lewis rats that were 8 weeks old were purchased from Charles River laboratories, maintained at the Experimental Animal Facility of Maastricht University in a temperature- and humidity-controlled environment subjected to a 12 h light/dark cycle and free access to food and drinking water. All animal experiments were conducted in compliance with the Declaration of Helsinki and Dutch regulations, with approval by the Dierexperimentencommissie (DEC) and the Centrale Commissie Dierproeven (CCD) (project license numbers AVD107002016781 and AVD10700202115692).

### 4.2. Isolation and Treatment of Adult Rat Cardiomyocytes

Rats were anesthetized intraperitoneally with pentobarbital (200 mg/kg), after which the hearts were rapidly removed. Adult rat cardiomyocytes (aRCMs) were isolated by Langendorff perfusion as previously described [50], seeded on laminin-coated plates (Sigma, St. Louis, MO, USA), and cultured in M199 culture medium (Gibco 31153, Thermo Fischer, Waltham, MA, USA) supplemented with 5 mM creatine, 3.2 mM carnitine, 3.1 mM taurine, 20 μM palmitate, palmitate/BSA ratio of 0.3:1 and 1% penicillin/streptomycin. For prevention experiments, cells were stimulated with or without 50 µM phenylephrine (PE, Sigma-Aldrich), with 2.5 µM MI14 (Cat. No. 5604, Tocris, Abingdon, UK), or with 50 µM PE and 2.5 µM MI14 together for 24 h. For treatment experiments, cells were stimulated with or without 50 µM PE for the first 24 h after which medium was refreshed and PE or PE with MI14 was added for the following 24 h. For adenoviral experiments, cells were transduced with AdGFP or AdPKD1 as described previously [17], with addition of MI14 during the last 24 h of culturing. During incubations, cells were maintained in an incubator (37 °C, 5% CO_2_). See Supplementary Materials Figure S1 for a schematic overview of these workflows.

### 4.3. Induced Pluripotent Stem Cell-Derived Cardiomyocytes

Induced pluripotent stem cell-derived cardiomyocytes (iPSC-CM) were created as previously described [33]. Cells were stimulated with or without 50 µM phenylephrine (PE, Sigma-Aldrich), with 2.5 µM MI14 (Tocris), or with 50 µM PE and 2.5 µM MI14 together for 24 h and maintained in an incubator (37 °C, 5% CO_2_).

### 4.4. RNA Isolation and RT-PCR

Total RNA was isolated using Trisure (Bioline, Waddinxveen, The Netherlands) and 1 µg cDNA was synthesized using the SensiFAST cDNA synthesis kit (Bioline). Relative gene expression was determined by RT-PCR using the Sensimix SYBR & Fluorence kit (Bioline) and the LightCycler 480 II real-time PCR System (Roche, Woerden, The Netherlands). The ∆∆CT method was used for quantification of BNP (5′→3′) (forward: AGGAGAGA CTTCGAAATTCCAAGA; reverse: CTAAAACAACCTCAGCCGTCA) and samples were normalized against the housekeeping genes GAPDH (forward: GGGTGTGAACCACGAGAAAT; reverse: ACTGTGGTCATGAGCCCTC) and Cyclophilin A (forward: TTCCTCCTTTCACAGAATTATTCCA; reverse: CCGCCAGTGCCATTATGG).

### 4.5. Cell Lysis and Western Blotting

Cell lysis was performed as described earlier [51]. Equal amounts of cell lysates were subjected to SDS-PAGE and blotted onto nitrocellulose or activated PVDF membranes. Membranes were blocked in a 5% non-fat dry milk solution with or without 0.5 mM sodium orthovanadate and 10 mM sodium fluoride for 1 h at RT, washed with TBS-T and then incubated overnight at 4 °C with primary antibodies (see Supplementary Materials Table S1). Thereafter, blots were washed, incubated with HRP-linked secondary antibodies (see Supplementary Materials Table S1) and washed again before visualization. Total expression of a protein target was detected on the same membrane after detection of the phosphorylated protein, by the same method as described above. Protein bands were detected using enhanced chemiluminescence (Bio-Rad, Veenendaal, The Netherlands) and quantified by densitometric analysis (Quantity one, Bio-Rad). Coomassie Brilliant Blue (CBB) staining, to determine total protein expression, was performed by washing PVDF membranes with parsing solution (0.19% glycine, 1% SDS, pH 2.0) for 2 h, staining with CBB for 5 min, de-staining with 50% methanol for 20 min and rinsing with distilled water for 15 min. Stained membranes were scanned, and Coomassie staining was measured with FIJI (version 1.54). Data was normalized to Coomassie staining or caveolin 3 expression.

### 4.6. Measurement of Substrate Uptake

Deoxyglucose and palmitate uptake was measured by washing the cardiomyocytes three times with uptake buffer (0.46% BSA and 1 mM CaCl_2_ in Modified Krebs Ringer (MKR) buffer) and adding a mixture of deoxyglucose (4 µM final), palmitate (20 µM final) and tracer amounts of [^3^H]-deoxyglucose (0.05 µCi/mL final, Perkin Elmer, Waltham, MA, USA) and [^14^C]-palmitate (0.011 µCi/mL final, Perkin Elmer). After 10 min incubation, the uptake was stopped by two washing steps with ice-cold wash buffer (0.1% BSA, 1 mM CaCl_2_, 0.2 M phloretin (Santa Cruz, Heidelberg, Germany) and 0.1% DMSO in MKR buffer). Cells were lysed in 1 M NaOH, and radioactivity was measured via liquid scintillation counting with a β-counter (Tri-Carb 2910 TR, Perkin Elmer). Data was normalized to total protein content, as determined by Bradford assay. As positive control, 30 min incubation with 100 nM insulin (Sigma I-5500) was used, and a sample without incubation time was used as negative control.

### 4.7. Measurement of Phenylalanine Incorporation

To assess protein synthesis rates, cultured cells were incubated with [^14^C]-phenylalanine (0.1 µCi/mL final, Perkin Elmer) under the examined conditions for 24 h. Then cells were washed three times with ice-cold PBS and proteins were precipitated by 10% trichloroacetic acid overnight at 4 °C. The samples were then rinsed two times with 95% ethanol, dissolved in 1 M NaOH containing 0.1% SDS and radioactivity was measured via liquid scintillation counting with a β-counter (Tri-Carb 2910 TR, Perkin Elmer). As negative controls, a sample without cells and a sample without incubation time were included.

### 4.8. Measurement of Contractile Function and Cardiomyocyte Size

Contractile properties of cardiomyocytes were analyzed under 30 V 1 Hz electric field stimulation. Recordings of contracting cells were made by bright-field microscopy and analyzed with FIJI (version 1.54). In more detail, a 6-well plate containing the cells with electrodes coupled on top was placed on a heating stage (Tokai Hit, Bala Cynwyd, PA USA) set at 39 °C on top of the microscope stage of an inverted microscope (CK2, Olympus, Hoofddorp, the Netherlands). A camera (MikroCamII, Bresser GmbH, Rhede, Germany) recorded the contraction videos with 60 fps at 1824 × 1216 pixels with a 10× objective. Field of view was 1404 × 936 μm^2^ with 3–8 cells visible in each video. Cells that were overlapping, not contracting, exhibiting apoptosis or not fully in view were not analyzed. Videos were imported in FIJI and segmented using the intensity threshold method (default algorithm). Segmented images were subsequently processed with the Analyze particles function of FIJI to measure the area, minor and major axis of the cells in each frame. The Beat Analysis tool (https://martijnhoes.shinyapps.io/myomate (accessed on 22 September 2024)) was used to calculate cell shortening and fractional shortening. Sarcomere shortening, time to peak and decay time were measured using the video-based geometry system IonOptix (Milton, MA, USA) and IonWizard acquisition software (version 6.2), as previously described [39]. To determine cell area, length and width, video frames of relaxed cells were analyzed with FIJI (version 1.54), using the intensity threshold method and rotated rectangle functions to outline cells and the area and shape descriptors measurements as output parameters.

### 4.9. Measurement of Cell Viability

aRCM seeded in 96 well plates were treated as described earlier. Media were replaced by medium containing 0.5 mg/mL Thiazolyl Blue Tetrazolium Bromide (Sigma-Aldrich) and incubated for 4 h. To stop the reaction, medium was removed and the crystals dissolved in 100% DMSO. Absorbance was measured at 570 nm with the CLARIOstar Plus (software version 5.70R2) (BMG Labtech, Ortenberg, Germany).

### 4.10. Statistics

All data are presented as means ± SEM. Statistical analysis was conducted using ordinary one-way ANOVAs followed by post hoc analysis with Fisher’s LSD test (Prism 8, GraphPad Software, Inc., San Diego, CA, USA). *p*-values below 0.05 were considered statistically significant.

## 5. Conclusions

PI4KIIIβ is a suitable target and MI14 a useful compound to decrease enhanced glucose uptake in PE-treated cardiomyocytes for prevention and treatment of contractile dysfunction in hypertrophic cardiomyocytes from both rodent and human origin, while keeping hypertrophy aspects unaffected. Moreover, these beneficial effects of MI14 can be extended to other cardiomyocyte models of hypertrophy than PE stimulation, as MI14 also successfully inhibited the maladaptive increases in glucose uptake in cardiomyocytes with PKD1 overexpression, another model of cardiac hypertrophy [17]. Even more importantly, MI14 also demonstrated inhibition of PE-stimulated glucose uptake in human cardiomyocytes, thereby advocating this selective PI4KIIIβ inhibitor as a promising pharmacological compound to combat hypertrophic cardiomyopathy.

## Data Availability

The datasets used and/or analyzed during the current study are available from the corresponding author upon reasonable request.

## Supplementary Materials

The following supporting information can be downloaded at: https://www.mdpi.com/article/10.3390/ijms27020595/s1.
