# Supplementary material for: Phosphatidylinositol 4-Kinase IIIβ: A Therapeutic Target for Contractile Dysfunction in Hypertrophic Cardiomyocytes
Source: Int J Mol Sci. 2026 Jan 7;27(2):595. doi: 10.3390/ijms27020595 (PMC12841316; doi:10.3390/ijms27020595)
Supplement: Supplementary file 1 [file ijms-27-00595-s001.zip › ijms-3925817-supplementary.pdf]

Phosphatidylinositol 4-kinase IIIβ: a therapeutic target for contractile dysfunction in hypertrophic cardiomyocytes

Supplementary materials

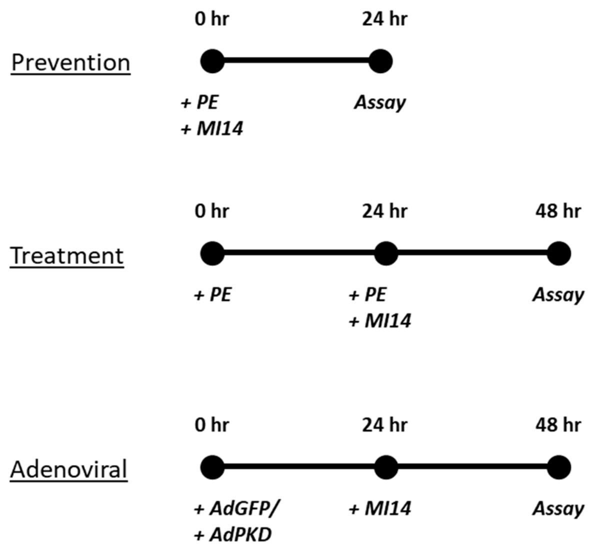

Figure S1 – Schematic overview of adult rat cardiomyocyte (aRCM) incubation workflow.

Table S1 – Detailed overview of antibody solutions for Western blotting.

Target	Primary antibody		Secondary antibody	
	Solution	Company	Solution	Company
**Cav3**	1:6000 in 1% nfdm	BD Transduction Laboratories BD610420	1:20,000 in TBS-T	Dako Cytomation P0161
**HDAC5**	1:1000 in 5% BSA/TBS-T	CST20458	1:2000 in 5% nfdm/TBS-T	CST7074
**mTOR**	1:1000 in 5% BSA/TBS-T	CST2972	1:2000 in 5% nfdm/TBS-T	CST7074
**pERK (Thr202/Tyr204)**	1:1000 in 5% BSA/TBS-T	CST9101	1:2000 in 5% nfdm/TBS-T	CST7074
**pHDAC5 (Ser498)**	1:1000 in 5% BSA/TBS-T	ab47283	1:9000 in TBS-T / 1:2000 in 5% nfdm/TBS-T	Jackson Immuno Laboratories Inc 711-035-152 / CST7074
**PKD**	1:1000 in 5% BSA/TBS-T	CST2052	1:2000 in 5% nfdm/TBS-T	CST7074
**PLN**	1:1000 in 5% BSA/TBS-T	CST14562	1:2000 in 5% nfdm/TBS-T	CST7074
**pmTOR (Ser2448)**	1:1000 in 5% BSA/TBS-T	CST2971	1:2000 in 5% nfdm/TBS-T	CST7074
**pPKD (Ser916)**	1:500 in 5% BSA/TBS-T	CST2051	1:2000 in 5% nfdm/TBS-T	CST7074
**pPLN (Ser16)**	1:1000 in 5% BSA/TBS-T	ab15000	1:2000 in 5% nfdm/TBS-T	CST7074
**pTnI (Ser23/24)**	1:1000 in 5% BSA/TBS-T	CST4004	1:2000 in 5% nfdm/TBS-T	CST7074

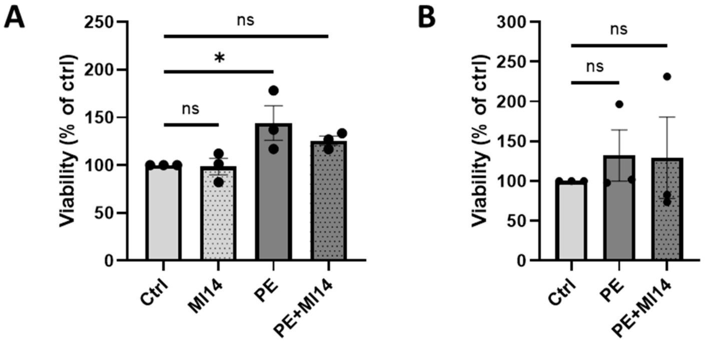

Figure S2 – Cell viability is not negatively affected by PE or MI14 incubation. Cell viability as assessed by MTT assay after **(A)** prevention and **(B)** treatment incubation timelines (*n*=3). Data is reported as percentage of control ± SEM. *P<0.05. PE, phenylephrine

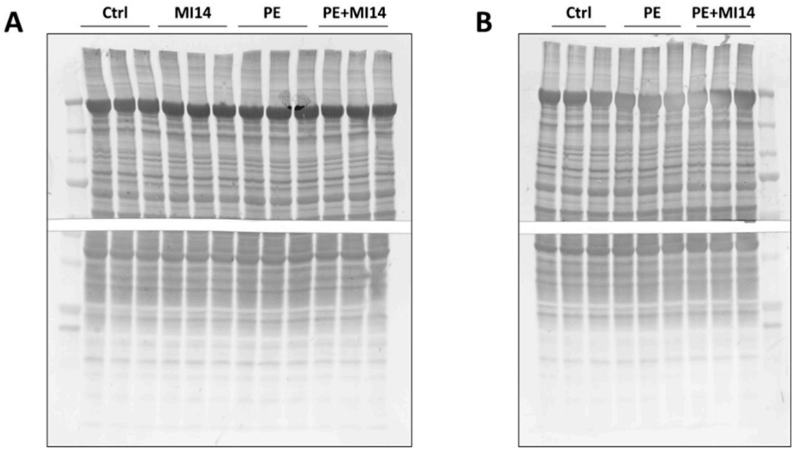

Figure S3 – Representative Coomassie images. Coomassie blots corresponding to the Western blots of the **(A)** prevention and **(B)** treatment experiments. PE, phenylephrine

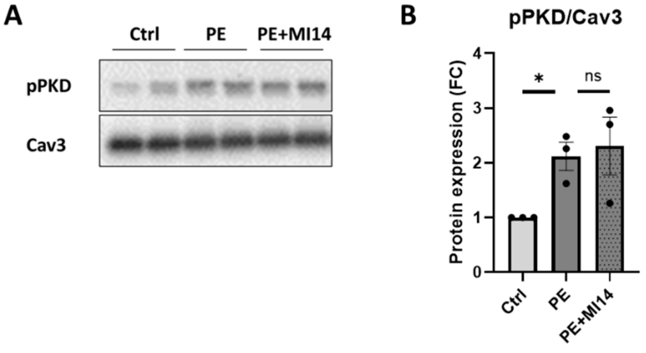

Figure S4 – Short-term (15 min) PI4KIIIβ inhibition by MI14 does not affect PE-induced PKD phosphorylation. (A) Representative Western blots of phosphorylated PKD and caveolin 3. **(B)** Quantification of target protein normalized to caveolin 3 (*n*=3). Data is reported as fold change of control ± SEM. *P<0.05. Cav3, caveolin 3; PE, phenylephrine; PKD, protein kinase D1.

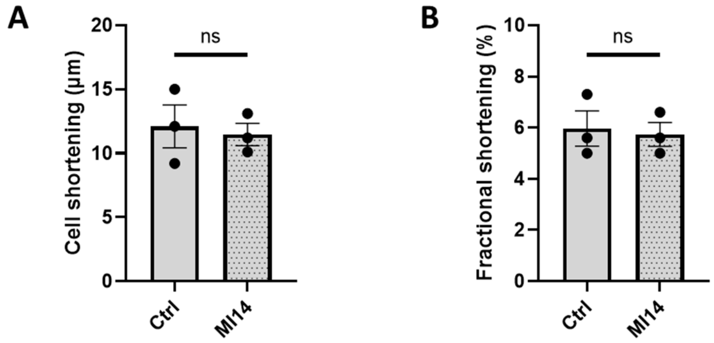

Figure S5 – MI14 does not affect basal contractile function. (A) Cell shortening and **(B)** fractional shortening of aRCM (*n*=3). Data is reported as mean ± SEM.

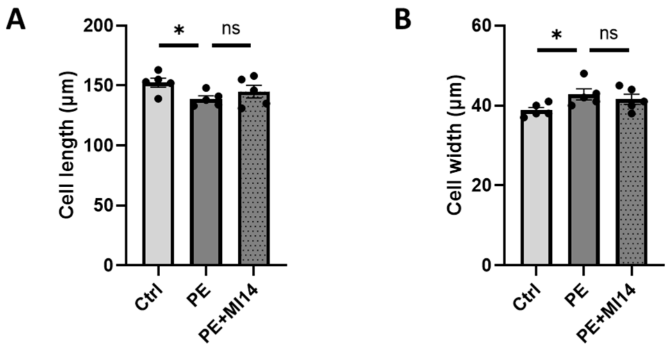

Figure S6 – MI14 does not affect PE-induced alterations in cardiomyocyte dimensions. Cardiomyocyte dimensions expressed in **(A)** cell length and **(B)** cell width (*n*=5). Data is reported as mean ± SEM. *P<0.05. PE, phenylephrine
